# Endothelin-1 from prostate cancer cells is enhanced by bone contact which blocks osteoclastic bone resorption

**DOI:** 10.1054/bjoc.2000.1261

**Published:** 2000-07-03

**Authors:** J W Chiao, B S Moonga, Y M Yang, R Kancherla, A Mittelman, J R Wu-Wong, T Ahmed

**Affiliations:** Department of Medicine, New York Medical College Valhalla, New York, 10595, USA; Abbott Laboratories, Abbott Park, Illinois, 60064–3500, USA

**Keywords:** prostate cancer, endothelin-1, bone lesions

## Abstract

The causes for the propensity of metastasized prostate cancer cells to grow in bone and to induce osteoblastic lesions remain unresolved. Co-culture of human prostate cancer cell lines with bone slices was determined to increase the level of endothelin-1 (ET-1) mRNA and its production. ET-1 is an ejaculate protein that also stimulates osteoblasts. Osteoclastic bone resorption was significantly blocked by the presence of androgen-independent prostate cancer cells in a dose-dependent manner as that of synthetic ET-1. The inhibition could be neutralized by specific ET-1 antibody, indicating the association of prostate cancer-derived ET-1 with inhibition of bone resorption. The combined ET-1 activity on osteoclasts and osteoblasts disrupts bone remodelling. ET-1 production is also elevated in the presence of prostate-specific antigen (PSA). ET-1 in turn enhances DNA synthesis of prostate cancer cells. Interactions among cancer cells, bone, ET-1 and PSA may be critical in cancer growth and lesions in bone. © 2000 Cancer Research Campaign


*British Journal of Cancer* (2000) **83**(3), 360–365
© 2000 Cancer Research Campaign
doi: 10.1054/ bjoc.2000.1261, available online at http://www.idealibrary.com on IDEAL®

### Endothelin-1 from prostate cancer cells is enhanced by bone contact which blocks osteoclastic bone resorption

**JW Chiao[1], BS Moonga[1], YM Yang[1], R Kancherla[1], A Mittelman[1], JR Wu-Wong[2] and T Ahmed[1]**

[1]Department of Medicine, New York Medical College Valhalla, New York 10595, USA; [2]Abbott Laboratories, Abbott Park, Illinois 60064–3500, USA

**Summary** The causes for the propensity of metastasized prostate cancer cells to grow in bone and to induce osteoblastic lesions remain unresolved. Co-culture of human prostate cancer cell lines with bone slices was determined to increase the level of endothelin-1 (ET-1) mRNA and its production. ET-1 is an ejaculate protein that also stimulates osteoblasts. Osteoclastic bone resorption was significantly blocked by the presence of androgen-independent prostate cancer cells in a dose-dependent manner as that of synthetic ET-1. The inhibition could be neutralized by specific ET-1 antibody, indicating the association of prostate cancer-derived ET-1 with inhibition of bone resorption. The combined ET-1 activity on osteoclasts and osteoblasts disrupts bone remodelling. ET-1 production is also elevated in the presence of prostate-specific antigen (PSA). ET-1 in turn enhances DNA synthesis of prostate cancer cells. Interactions among cancer cells, bone, ET-1 and PSA may be critical in cancer growth and lesions in bone. © 2000 Cancer Research Campaign

**Keywords**: prostate cancer; endothelin-1; bone lesions

Bone is one of the most common sites of prostate cancer metastases. In approximately 95% of cases the cancer promotes new bone formation, resulting in osteoblastic lesions (Sharp and McDonald, 1942; Koutsilieris, 1993). Infiltrating prostate cancer cells are believed to cause a sclerotic reaction and impaired mineralization (Charbon et al, 1983). The remaining 5% of bone metastases are a mix of osteoblastic and osteolytic lesions which are usually associated with the terminal stages of the disease. The causes for the propensity of prostate cancer to grow in bone and form osteoblastic lesions remain unresolved.

Previous investigations of osteoblastic lesions have focused on the prostatic cancer cell effect on osteoblasts and the identification of mitogenic growth factors produced. Several peptides including an amino-terminal fragment of urokinase from human prostate cancer PC-3 cells were identified as the osteoblastic factor (Rabbani et al, 1990). Increased osteoblast proliferation has been described to result in the inhibition of differentiation, collagen synthesis and mineralization (Martinez et al, 1996; Santibanez et al, 1996). Since normal bone remodelling requires the balanced activity of osteoclasts in resorbing and osteoblasts in building the bone, altered function of either could affect bone remodelling. To investigate progressive bone destruction in prostate cancer it is necessary to understand the biology of osteoclasts in the pathogenic process. In this regard we have examined endothelin-1 (ET-1), which may have a potential role in bone metabolism. ET-1 is an ejaculate peptide with 21 amino-acid residues (Casey et al, 1992). ET-1 is produced by a variety of tissues including endothelial cells (Yanagisawa, 1994) and prostate cancer cells (Nelson et al, 1995). ET-1 has been proposed to contribute to the osteoblastic response. This includes the modulation of the mRNA expression of osteopontin and osteocalcin which are phenotype-related gene products of osteoblasts (Shioide and Noda, 1993) and the stimulation of new bone formation in a mouse model (Nelson et al, 1999). Although ET-1 from bone marrow was previously suggested as an important regulator for osteoclasts, that activity was not linked to prostate cancer cells. In this report we have investigated the osteoclast-inhibiting activity of ET-1 derived from prostate cancer cells. There is evidence that ET-1 is a major factor responsible for the prostate cancer cell-mediated inhibition of osteoclastic bone resorption. Additionally, ET-1 level in prostate cancer cells is enhanced by bone or PSA stimulation.

*Received 7 December 1999*
*Revised 24 March 2000*
*Accepted 3 April 2000*

*Correspondence to:* JW Chiao

### MATERIAL AND METHODS

### Cell lines and ET-1 measurement

Androgen-independent human prostate cancer cell lines DU-145, PC-3, and JCA-1 (Muraki et al, 1990) and androgen-dependent LNCaP cell lines (Horoszewicz et al, 1983) were maintained in RPMI-1640 medium with 7% heat-inactivated fetal calf serum (FCS). These cell lines were also maintained instead with 7% heat-inactivated FCS removed of steroids with charcoal stripping (HyClone Lab, Inc, Logan, UT, USA) to avoid possible interference in cell growth. Some of these cells were co-cultured with devitalized bovine cortical bone slices pre-wetted with RPMI-1640 medium (Dempster et al, 1987). The bottom of the culture vessels were covered with bone slices, $4.4 \times 4.4$ mm pieces were placed in each of the flat bottom wells of the 96-well plates prior to addition of cells. During the culture period aliquots of the supernatants were collected for the measurement of ET-1 level by a sandwich immunoassay (R and D System, Minneapolis, MN, USA) for quantitation of human ET-1 in fluid samples.

Some cell cultures at $1.5 \times 10^5$ cells $ml^{-1}$ in RPMI-1640 and 7% charcoal-stripped FCS were exposed to purified prostate-specific antigen (PSA) (Chemicon International, Inc, Temecula, CA, USA) prepared in RPMI-1640 medium at various concentrations for

2 days. The culture supernatants with or without PSA were collected and assayed for ET-1 levels by ELISA.

### RT-PCR and Northern blot analysis

Total RNAs were extracted from prostate cancer cells with or without exposure to bone slices for 1–2 days using a total RNA isolation kit Ultraspec-II RNA (Biotech Lab Inc, Houston, TX, USA). A Gene Amp RNA PCR Kit (Perkin Elmer, Foster City, CA, USA) was employed for reverse transcription using oligo dT and AMV reverse transcriptase. The cDNAs were used as templates for PCR amplification. The amplification was performed with 35 cycles of 1 min denaturing at 94°C, 1 min annealing at 54°C, and 1 min elongation at 72°C. The specific primers for ET-1 are sense 5′-TTA AAG GGC ACT TGG GCT GA-3′, antisense 5′-AAG ATG ATT TGA CGC TGT TT-3′, representing positions 202–222/885–905 of endothelin-1 sequence (Genbank: accession S56805). The expected size of PCR products is 703 bp (Nelson et al, 1995). The primers for actin are 5′CCT CGC CTT TGC CGA TCC-3′, antisense 5′-GGA TCT TCA TGA GGT AGT CAG TC-3′. The predicted amplicon is 626 bp (Raff et al, 1997). The presence of the PCR product was ascertained by electrophoresis on 7.5% PAGE after staining with ethidium bromide.

Electrophoresis and hybridization of Northern blot analyses were performed by standard procedures. Blots were washed twice at 65°C in 2 × SSC and 0.1% SDS for 15 min, followed by detection of autoradiography. Probes for ET-1 were labelled with $\gamma^{32}$P-dCTP. The amount of RNA loaded was monitored with 28 S and 18 S ribosomal RNA stained by ethidium bromide.

### Cell proliferation

Prostate cancer cell lines at $1.5 \times 10^5$ cells ml$^{-1}$ in RPMI-1640 medium with 7% charcoal-stripped FCS were exposed to synthetic ET-1 prepared in RPMI-1640 medium at various concentrations. During the culture period, each culture was labelled with 2 μCi ml$^{-1}$ of [$^3$H]thymidine followed with trypsination and scintillation counting (Abolhassani and Chiao, 1995). At least triplicate experiments were performed for each data point and the mean count per minute was presented.

### Bone resorption assay

Bone resorption was quantified using the disaggregated osteoclast resorption assay originated by Boyde et al (1984) and Chambers et al (1984) and performed as described by Dempster et al (1987). Briefly, osteoclasts were isolated from the long bones of neonatal rats onto devitalized 4.4 × 4.4 mm bovine cortical bone slices pre-wetted with medium 199 + Hanks' salts. The slices were rinsed to remove non-adherent cells and transferred to medium 199 containing Earle's salts, sodium bicarbonate and 10% heat-inactivated FCS containing vehicle alone or ET-1 prepared in the same medium. Some bone-resorption cultures were added with prostate cancer cell lines prepared in the same medium 199 approximately 18 h prior to the addition of isolated osteoclasts. Some of these cultures were further supplemented with a rabbit antiserum with specificity against human ET-1 (Abbott Laboratories, Abbott City, IL, USA) at a final dilution of 1:1000. The antiserum supplemented alone in the culture was employed as a control.

After incubation the bone slices were fixed and stained for tartrate-resistant acid phosphatase. The number of multinucleated osteoclasts were counted on 12 replicate slices per treatment. Cells were stripped from the slices by sonication in 0.25 M $NH_4OH$, and the number of resorption pits counted in the light microscope on 12 replicates per treatment after staining in toluidine blue. Osteoclast viability was determined using the trypan blue exclusion method.

### Statistical analysis

A repeated measures analysis of variance was used to estimate the within-subject effect of treatment (SAS Proc GLM, SAS Institute, Cary, NC, USA). Preplanned contrasts compared the effect of each treatment to the control and used a type I Bonferonni correction; comparison-wise error rate was used to maintain an overall error rate of 5%.

### RESULTS

### ET-1 increases after bone stimulation

The quantity of ET-1 in culture supernatants of prostate cancer cell lines with or without co-culture with devitalized bovine bone slices was determined by an ELISA procedure. Figure 1 shows that ET-1 could be detected in the supernatant from unstimulated cultures without bone slices. The culture media used as background lacked ET-1. After co-culturing with bone slices, there was a significant increase of ET-1 in prostate cancer cell lines (Figure 1). ET-1 increase could be detected as early as 6 h after the initiation of cultures. The magnitude of the ET-1 enhancement varied among cell lines. ET-1 from androgen-independent cells DU-145, PC-3 or JCA-1 increased from a mean of 4, 3 or 5 pg ml$^{-1}$ to 8, 6 or 7 pg ml$^{-1}$ per $10^5$ cells by 24 h; respectively, representing approximately 40–100% increases (Figure 1). The androgen-dependent LNCaP cells had a near background level of ET-1 without bone culturing and the increase of ET-1 after exposure to bone was relatively minor. LNCaP cells maintained in either regular FCS or in steroid-depleted FCS showed no difference in this regard. Culture supernatant from bone slices without cells was used as a background control, which had no detectable ET-1. These results suggest that an increase of ET-1 quantity was the result of stimulation by bone.

By RT-PCR amplification and Northern RNA blot analysis a significantly enhanced level of ET-1 mRNA was detected with these prostate cancer cell lines after co-culturing with bone slices, as compared to those without bone. Figure 2 shows the increase by RT-PCR with representative cell line DU-145. An autoradiograph of Northern blot analysis is shown in Figure 2B, revealing an expected 2.3 kb band (Bagnato et al, 1999), demonstrating the presence of ET-1 mRNA in untreated cells and the increase after bone exposure.

### Inhibition of osteoclast bone resorption

Cells from human prostate cancer cell lines were added to bone resorption cultures containing isolated osteoclasts, to evaluate their effect on osteoclastic bone resorption. The presence of androgen-independent PC-3, JCA-1 or DU-145 cells but not androgen-dependent LNCaP cells caused significant ($P < 0.05$)

*© 2000 Cancer Research Campaign* 

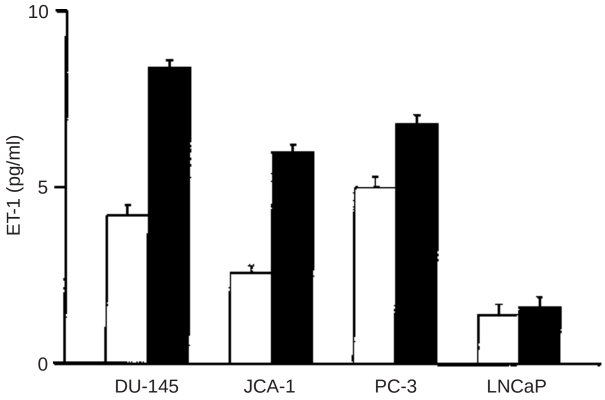

**Figure 1** Quantification of ET-1 (pg $ml^{-1}$) in the culture supernatants from human prostate cancer cell lines co-cultured with (black) or without (white) devitalized bovine bone slices by ELISA. Aliquots of 1-day culture supernatants were collected for the measurement. Graphs recorded the ET-1 quantities per $1.5 \times 10^5$ cells of androgen-independent prostate cell lines DU-145, PC-3, JCA-1 and androgen-dependent LNCaP cells. Each cell line was maintained in medium RPMI-1640 with 7% charcoal-stripped FCS. No ET-1 was detected in culture media or bone slice conditioned medium, negative controls. Vertical bars indicate median values ± SEM.

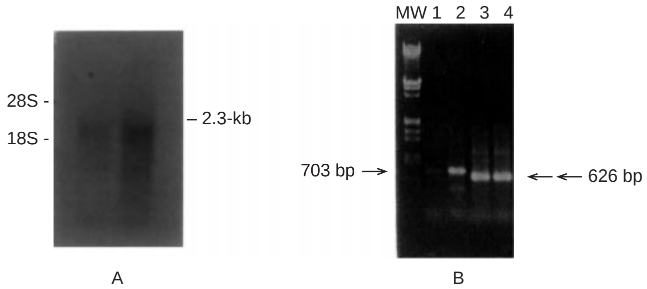

**Figure 2** Northern blot analysis (**A**) and RT-PCR amplification (**B**) show increased expression of ET-1 in prostate cancer cells induced by bone. Left lane in (**A**) Northern blot analysis indicates ET-1 from untreated prostate cancer cells DU-145 cells at 2.3 kb position while right lane ET-1 from cells co-cultured with bone slices. DU-145 cells were cultured with or without bone for 1 day. Lanes 1 and 2 (**B**) are 703 bp RT-PCR products of ET-1 and lanes 3 and 4 are 626 bp RT-PCR products of control gene actin. Lanes 1 and 3 are from untreated DU-145 and lanes 2 and 4 are cells co-cultured with bone slices for 1 day. MW is molecular weight marker of λ DNA digested by Hind III and EcoRI.

inhibition. Figure 3A shows that approximately 50% reduction of bone resorption, as indicated by the number of resorption pits per bone slice, could be achieved with DU-145 cells used at $10^5$ cells $ml^{-1}$. This condition for mid-point inhibition was chosen for subsequent analyses with a specific anti-human ET-1 antibody. The addition of the antibody with DU-145 cells quantitatively negated the DU-145-mediated inhibition (Figure 3A). A non-specific Ig preparation used as a control showed no effect. The antibody was also added to bone resorption cultures with LNCaP cells but showed no effect, since LNCaP cells did not have an significant effect on bone resorption (Figure 4A). The antibody used alone in osteoclast cultures as a control also had no effect (Figure 3A). There was no evidence of a lytic effect on osteoclasts by any of the cell lines or in the antibody studies; the number of osteoclasts per slice at the end of the experiments remained unchanged from control values (Figures 3B and 4B). Other controls include the addition of prostate cancer cells to bone resorption cultures without osteoclasts, which showed no bone resorption, and the exposure of prostatic cells to the antibody, which revealed no cytolysis.

Figure 3A shows further that exposure to synthetic ET-1 at 10 nM could inhibit approximately 50% bone resorption, duplicating the effect of DU-145 cells.

Supplementation of ET-1 together with the anti-ET-1 antibody completely blocked ET-1 activity (Figures 3A and 4A), indicating a direct ET-1 activity on osteoclasts. Figure 3A also shows that the combination of DU-145 and ET-1 used in the bone resorption assay achieved a level of inhibition which was slightly more than either used separately. There was no evidence of a toxic effect of ET-1; the number of osteoclasts per slice at the end of the experiments remained unchanged from control values (Figures 3B and 4B). Additionally, the proportion of osteoclasts excluding trypan blue was the same in the presence or absence of ET-1. These results suggest that ET-1 derived from DU-145 cells represents the major factor responsible for modulating the osteoclastic bone resorption. In contrast, androgen-dependent LNCaP cells may not have a sufficient quantity of ET-1 to affect the osteoclastic function.

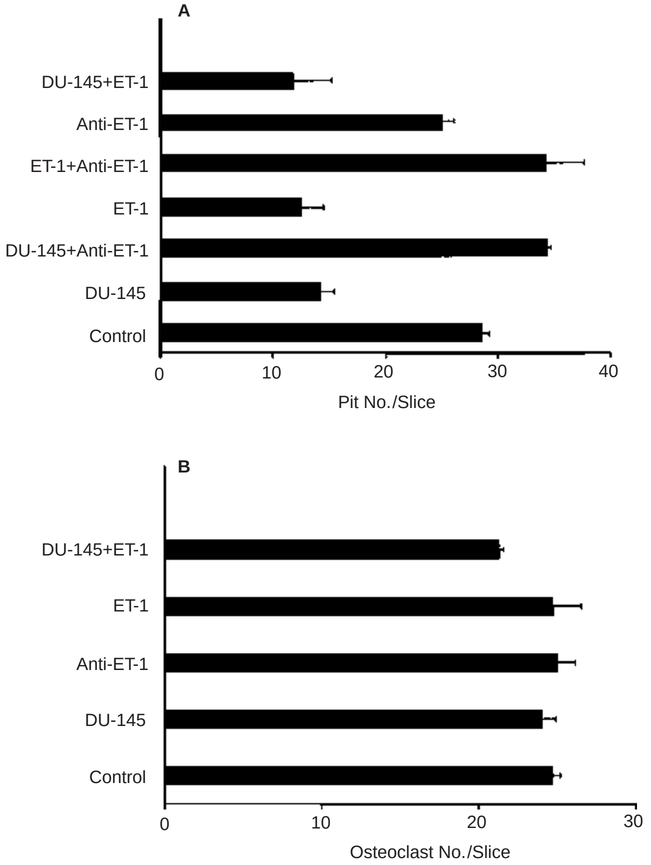

**Figure 3** Effect of human androgen-independent prostate cancer cell line DU-145 ($1 \times 10^5$ cells $ml^{-1}$) or synthetic ET-1 (10 nM) on bone resorption (**A**) and on isolated rat osteoclasts (**B**). Control indicates resorption by isolated osteoclasts in medium 199. Horizontal bar values are means ± SEM.

© 2000 Cancer Research Campaign

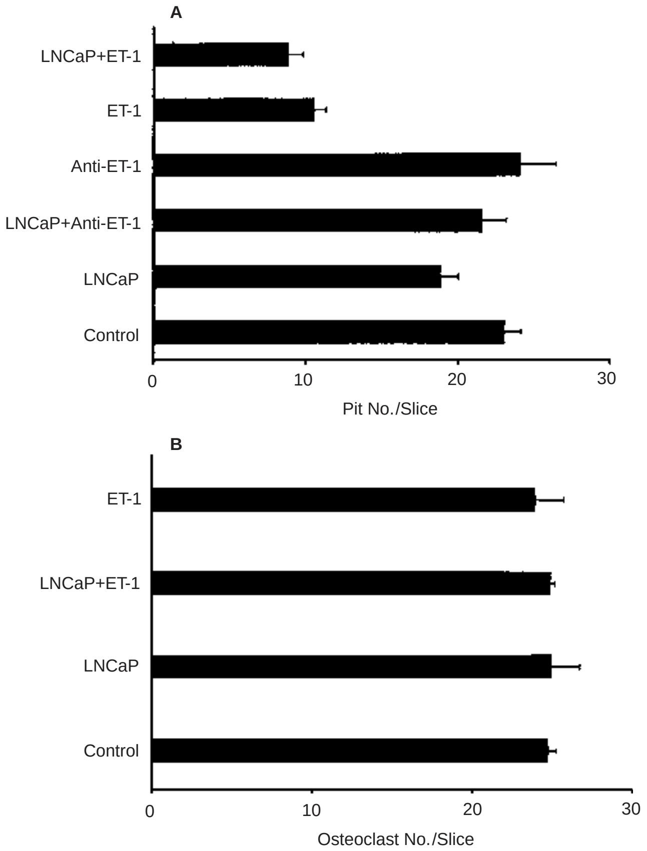

**Figure 4** Effect of human androgen-dependent prostate cancer cells LNCaP cells ($1 \times 10^5$ cells $ml^{-1}$) on bone resorption (**A**) and on isolated rat osteoclasts (**B**). Synthetic ET-1 used at 10 nM was used as a positive control of inhibition of bone resorption. Resorption by isolated osteoclasts in medium 199 was used as a control. Horizontal bar values are means ± SEM.

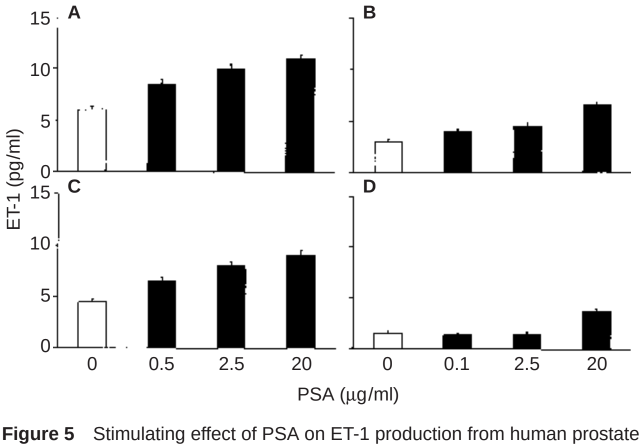

**Figure 5** Stimulating effect of PSA on ET-1 production from human prostate cancer cell lines maintained in RPMI-1640 with 7% charcoal-stripped FCS for 2 days. Graphs recorded the ET-1 quantities in the culture supernatants from PC-3 (**A**), JCA-1 (**B**), DU-145 (**C**), and LNCaP cells (**D**) as detected by ELISA. Vertical bars indicate means ± SEM.

### PSA stimulates ET-1 production

The effect of PSA on the production of ET-1 from prostatic cells was analysed. Prostate cell lines were supplemented with purified

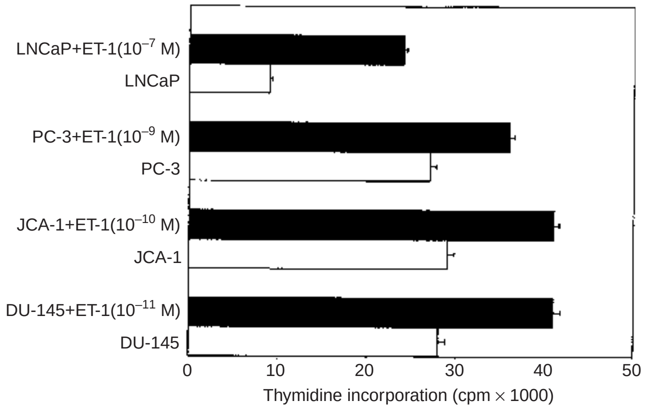

**Figure 6** Stimulating effect of ET-1 on proliferation of human prostate cancer cell lines as determined by [$^3$H]thymidine incorporation. Graph shows maximum enhancement of proliferation of DU-145, JCA-1, and PC-3 cells after 1 day ET-1 exposure, and LNCaP cells after 2 days. Horizontal bars represent means ± SEM of three experiments.

PSA at various concentrations and the ET-1 quantity in the culture supernatant was determined by ELISA. Medium controls containing PSA at the same concentrations showed no detectable ET-1. Figure 5 shows a PSA dose-related stimulatory effect on ET-1 production that was detected for DU-145, PC-3, JCA-1 and LNCaP cells. At PSA concentrations of 2.5–20 µg $ml^{-1}$, a graduate elevation of ET-1 with a maximum increase of 50% was detected for JCA-1, PC-3 and DU-145 cells. For LNCaP cells, the stimulatory effect for ET-1 production was only found at a higher dose of 20 µg $ml^{-1}$ PSA but not at lower dosages (Figure 5).

### ET-1 stimulates DNA synthesis

Experiments were performed to determine the modulating effect of ET-1 on the proliferation of prostate cancer cells. Each cell line in steroid-depleted FCS medium was supplemented with exogenous ET-1 at various concentrations and proliferation was assayed with [$^3$H]thymidine incorporation. ET-1 has a time- and dose- dependent effect on the increase of DNA synthesis of prostate cancer cells within a dose range of $1 \times 10^{-12}$–$1 \times 10^{-7}$ M. A moderate stimulatory effect was consistently observed after 1-day incubation. Figure 6 shows the maximum activity seen with various ET-1 dosages such that approximately 32%, 29% and 25% increase of DNA synthesis was observed with DU-145, JCA-1 and PC-3 cells, respectively. In contrast to these androgen-independent cells, androgen-dependent LNCaP cells had an approximately 10–16% reduction of DNA synthesis after a 1-day exposure to ET-1, and enhancement of approximately 62% after 2 days (Figure 6).

### DISCUSSION

Utilizing a co-culture method consisting of prostate cancer cells and bone slices, this report demonstrated that human prostate cancer cells produce increased quantities of ET-1 after contacting bone. The increase of ET-1 in the cultures was determined with an ELISA procedure, while the elevation of mRNA level was shown by RT-PCR and Northern blot analysis. The association of prostate cancer cell-derived ET-1 with bone resorption was clarified.

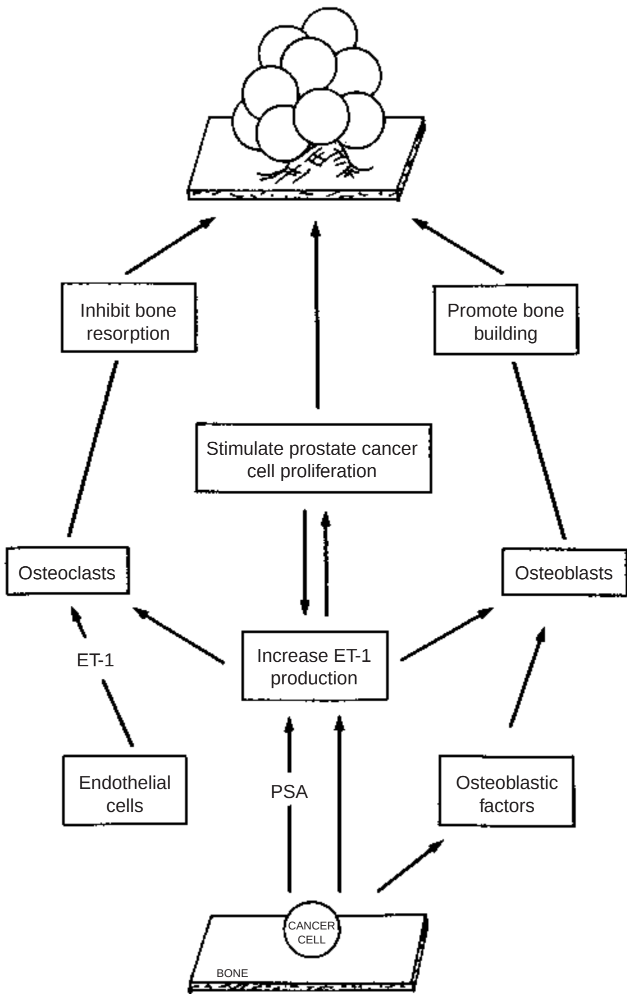

**Figure 7** Diagram showing the interactions of prostate cancer cells, PSA and ET-1 from cancer cells and endothelial cells in bone to promote cancer cell growth and bone damage.

Osteoclastic bone resorption was inhibited by the presence of prostate cancer cells to a degree similar to inhibition by ET-1. The inhibition could be neutralized with a specific anti-ET-1 antibody. The degree of antibody blocking was nearly complete, suggesting that ET-1 is the major factor from prostate cancer cells responsible for the inhibition of osteoclastic bone resorption. The antibody most likely neutralized ET-1 after its release from cells, as we have reported previously (Moonga and Chiao, 1998) that the activity inhibiting osteoclastic bone resorption from prostate cancer cells was secreted to the supernatants before interacting with osteoclasts. ET-1 from bone marrow cells as a regulator for osteoclasts was previously reported (Towhidul et al, 1992), but no reference was made linking this ET-1 function with prostate cancer cells. Our experimental results support the interpretation that prostate cancer cells in bone are induced to produce more ET-1 which stimulates osteoblasts and also regulates osteoclast function. The up-regulated ET-1 could disrupt the balance of bone remodelling. The direction of the abnormal bone remodelling, such as increased bone formation and resorption, may further depend on the balance of ET-1 activity on osteoblasts and osteoclasts. It is conceivable that other cytokines in bone regulation are important in this regard as well, and could also influence the ET-1 activity. The expression of these cytokine genes could be similarly affected by the cancer cells and ET-1. However, this needs to be defined. The combined effects of the altered genes and cytokine activities are therefore postulated as a mechanism in the bone damage.

Compared to the androgen-independent prostate cancer cell lines, androgen-dependent LNCaP cells do not have a significant effect on bone resorption. LNCaP cells whether unstimulated or exposed to bone had a level of ET-1 which was comparatively lower than the other cell lines on a per-cell basis. ET-1 in LNCaP cells can only be stimulated with PSA using much higher quantities than for androgen-independent cancer cells. These results indicate that LNCaP cells may not actively produce ET-1 as a major contributor affecting bone remodelling. Whether androgen-unresponsive prostate cancer cells represent the prime cell type causing osteoblastic bone lesions, and whether androgen-responsive cells are only involved in the presence of highly elevated PSA, is currently being investigated.

In addition to stimulation by bone, ET-1 levels in prostate cancer cells could also be elevated by exposure to PSA in a dose-dependent manner. This finding reveals a regulatory activity of PSA which has not been previously described. PSA has been reported to have a direct stimulatory effect on the proliferation of prostate cancer cells (Wang et al, 1996). This finding suggests that PSA enhances ET-1 production, with ET-1 subsequently stimulating cell proliferation. The observation supports the hypothesis that when prostate cancer cells metastasize to bone, they are stimulated by bone to produce more ET-1, which in combination with ET-1 from endothelial cells in bone marrow provides stimulation for the growth of tumour cells (Figure 7). The cancer cells in turn provide more ET-1 and PSA, promoting their own proliferation and also affecting the activities of osteoblasts and osteoclasts and hence bone remodeling. Figure 7 illustrates the role of ET-1 and PSA in perpetuating the interactions between cancer and bone cells to cause cancer growth and bone damage.

### REFERENCES

Abolhassani M and Chiao JW (1995) Antiproliferative effect of a prostate cell-derived activity on the human androgen-dependent prostatic carcinoma cell line LNCaP. *J Interferon Cytokine Res* **15**: 179–185

Bagnato A, et al (1999) Expression of endothelin 1 and endothelin A receptor in ovarian carcinoma: evidence for an autocrine role in tumour growth. *Cancer Res* **59**: 720–727

Boyde A, Ali NH and Jones SI (1984) Resorption of dentine by isolated osteoclasts in vitro. *Br Dent J* **156**: 216–220

Casey ML, Byrd W and MacDonald P (1992) Massive amounts of immunoreactive endothelin in human seminal fluid. *J Clin Endocrinol Metab* **74**: 223–228

Chambers TJ, et al (1984) Resorption of bone by isolated rabbit osteoclasts. *J Cell Sci* **66**: 383–399

Charbon SA, et al (1983) Histomorphometric analysis of sclerotic bone metastases from prostatic carcinoma with special reference to osteomalacia. *Cancer* **51**: 918–927

Dempster DW, Marrills RJ, Herbert W and Arnett TR (1987) Biological activity of chicken calcitonin: effects on neonatal rat and embryonic chick osteoclasts. *J Bone Mineral Res* **2**: 443–448

Horoszewicz JS, et al (1983) LNCaP model of human prostatic carcinoma. *Cancer Res* **43**: 1809–1818

Koutsilieris M et al (1987) Characteristics of prostate-derived growth factors for cells of the asteoblast phenotype. *J Chem Invest* **80**: 941–948

Martinez J, Silva S and Santibanez JF (1996) Prostate-derived soluble factors block osteoblast differentiation in culture. *J Cell Biochem* **61**: 18–25

 *© 2000 Cancer Research Campaign*

Moonga BS and Chiao JW (1998) Detection of an inhibiting activity for osteoclastic bone resorption from human prostatic cancer cells. *Cancer Lett* **123**: 15–20

Muraki J, et al (1990) Establishment of a new human prostatic cancer cell line (JCA-1). *Urology* **36**: 79–84

Nelson JB, et al (1999) New bone formation in an osteoblastic tumour model is increased by endothelin 1 over expression and decreased by endothelin A receptor. *Blockade Urology* **53**: 1063–1069

Nelson JB, et al (1995) Identification of endothelin-1 in the pathophysiology of metastatic adenocarcinoma of the prostate. *Nat Med* **1**: 944–949

Rabbani SA, et al (1990) An amino-terminal fragment of urokinase isolated from a prostate cancer cell line (PC-3) is mitogenic for osteoblast-like cells. *Biochem Biophys Res Commun* **173**: 1058–1064

Raff T, et al (1997) Design and testing of beta-actin primers for RT-PCR that do not co amplify processed pseudogenes. *Biotechnique* **23**: 456–460

Santibanez JF, Silva S and Martinez J (1996) Soluble factors produced by PC-3 prostate cells decrease collagen content and mineralization rate in fetal rat osteoblasts in culture. *Br J Cancer* **74**: 418–422

Sharp WS and McDonald JR (1942) Reaction of bone to metastasis from carcinoma of the breast and prostate. *Arch Pathol* **17**: 312–317

Shioide M and Noda M (1993) Endothelin modulates osteopontin and osteocalcin messenger RNA expression in rat osteoblastic osteosarcoma cells. *J Cell Biochem* **53**: 176–180

Towhidul ASM, et al (1992) Endothelin inhibits osteoclastic bone resorption by a direct effect on cell motility: implications for the vascular control of bone resorption. *Endocrinology* **130**: 3617–3624

Wang LG, Liu XM, Kreis W and Budman DR (1996) Involvement of prostate specific antigen in the stimulation of LNCaP cell growth. *Oncology Report* **3**: 911–917

Yanagisawa M (1994) The endothelin system. A new target for therapeutic intervention. *Circulation* **89**: 1320–1322

*© 2000 Cancer Research Campaign*
